# Mannose‐capped lipoarabinomannan‐induced B10 cells decrease severity of dextran sodium sulphate‐induced inflammatory bowel disease in mice

**DOI:** 10.1111/sji.12843

**Published:** 2019-11-24

**Authors:** Chun-Hui Yuan, Xin Li, Liang Luo, Ya-Ping Wang, Dong-Li Zhang, Kai‐Liang Zhou, Xiao‐Lian Zhang, Qin Pan

**Affiliations:** ^1^ Department of Immunology State Key Laboratory of Virology and Medical Research Institute Hubei Province Key Laboratory of Allergy and Immunology Wuhan University School of Basic Medical Sciences Wuhan China; ^2^ Department of Laboratory Medicine Wuhan Medical and Health Center for Women and Children Tongji Medical College Huazhong University of Science and Technology Wuhan China; ^3^ The Eighth Hospital of Wuhan Wuhan China

**Keywords:** B cells, IL‐10, inflammatory bowel disease, ManLAM

## Abstract

Inflammatory bowel disease (IBD) is a chronic, non‐specific, inflammatory gastrointestinal disease that mainly consists of Crohn's disease and ulcerative colitis. However, the aetiology and pathogenesis of IBD are still unclear. B10 (IL‐10 producing regulatory B) cells, a subset of regulatory B cells, are known to contribute to intestinal homeostasis and the aberrant frequency of B10 cells is associated with IBD. We have recently reported that B10 cells can be induced by ManLAM (mannose‐capped lipoarabinomannan), a major cell‐wall lipoglycan of *M tb* (*Mycobacterium tuberculosis*). In the current study, the ManLAM‐induced B10 cells were adoptively transferred into IL(interleukin)‐10^−/−^ mice and the roles of ManLAM‐induced B10 cells were investigated in DSS (dextran sodium sulphate)‐induced IBD model. ManLAM‐induced B10 cells decrease colitis severity in the mice. The B10 cells downregulate Th1 polarization in spleen and MLNs (mesenteric lymph nodes) of DSS‐treated mice. These results suggest that IL‐10 production by ManLAM‐treated B cells contributes to keeping the balance between CD4^+^ T cell subsets and protect mice from DSS‐induced IBD.

## 1 INTRODUCTION

B10 cells, a subset of regulatory B cells, are functionally characterized by their capacity to produce IL‐10.^1^ These B10 cells modulate immune response by activating regulatory T cells (Tregs), regulating Th1/Th2 cytokine balance and dendritic cell functions, downregulating the pro‐inflammatory network and suppressing T cell‐mediated autoimmunity.^2^ Aberrant frequencies of B10 cells have been reported in a variety of autoimmune and inflammation‐related diseases, indicating the important function of B10 cells to maintain immune homeostasis.^3^, ^4^

Inflammatory bowel disease, including Crohn's disease and ulcerative colitis, is a group of chronic inflammatory disorders characterized by epithelial barrier damage and disruption of immune homeostasis in the gastrointestinal tract. Its incidence is increasing globally; however, the precise aetiology remains unclear and a cure for IBD has yet to be discovered.^5^, ^6^ There is increasing evidence that they share in common disordered CD4^+^ T cell response, aberrant cytokine production and inflammation.^7^ Accumulating data from clinical and experimental studies has highlighted the important role of B10 cells existed in mesenteric lymph node and peritoneal cavity in the control of IBD.^8^, ^9^, ^10^, ^11^ B10 cells are also described with the ability to suppress disease progression by downregulating inflammatory cascades associated with IL‐1 upregulation and STAT3 activation in a mouse IBD model.^12^ However, it lacks the experimental assessment of B10 cells as candidate modulators for IBD treatment.

BCG (*Mycobacterium bovis* Bacillus Calmette‐Guérin) is a live attenuated vaccine against tuberculosis. As a strong inducer of Th1 type immunity, BCG has shown therapeutic effects on autoimmune diseases in murine studies, such as multiple sclerosis and insulin‐dependent diabetes.^13^ BCG has recently shown to reduce inflammation in murine IBD model by increasing the number of IL‐10‐producing Tregs.^14^ BCG given before 4 months of age may decrease the risk of IBD in people.^15^ We have previously reported that B10 cells have been induced by ManLAM (mannose‐capped lipoarabinomannan), a major cell‐wall lipoglycan of BCG and *M tb* (*Mycobacterium tuberculosis*).^16^ Therefore, we asked whether ManLAM‐induced B10 cells alleviated the inflammation and had the protective effects against IBD. In this study, the ManLAM‐induced B10 cells were transferred into IL‐10^−/−^ mice and the roles of B10 cells were investigated in DSS‐induced IBD model.

## 2 MATERIALS AND METHODS

### 2.1 Animals

IL10^−/−^ C57BL/6J mice were purchased from Nanjing biomedical institute of Nanjing University.^17^ WT (Wild type) C57BL/6J mice were purchased from the Animal Laboratory Center of Wuhan University, China. Mice used in the current study were female, 6‐8 weeks of age, weighing 18‐20 g. The mice were bred and maintained in the animal facilities of the Animal Laboratory Center of Wuhan University (Wuhan, China). The maintenance and care of mice complied with the guidelines of the University of Wuhan Ethic Committee.

### 2.2 ManLAM preparation

ManLAM was prepared from *M tb* H37Rv (ATCC strain 93009) or BCG (ATCC strain 35734) as previously described.^16^, ^18^ ManLAM was extracted from delipidated bacteria, purified by high‐performance liquid chromatography (HPLC) and identified as our previous reports.^16^, ^18^ Briefly, the bacteria were maintained on L‐J (Lowenstein‐Jensen) medium and were harvested while in log phase growth. The bacterial cells were delipidated using CHCl_3_: CH_3_OH (2:1, v/v) at 37°C for 12 hours. Then, the bacteria were delipidated by CHCl_3_: CH_3_OH:H_2_O (10:10:3, v/v/v) for an additional 12 hours. After drying the bacterial pellets, they were lysed with an ultrasonic disruptor in the buffer containing a protease inhibitor PMSF (#ST505, Beyotime Biotech, Haimen, China), DNase (#1121, BioFroxx, Hannover, Germany) and RNase (#1341, BioFroxx, Hannover, Germany) in PBS. Triton X‐114 (8% v/v) was added to the lysed cells and the solution mixed at 4°C overnight. After centrifugation at 27 000 g for 1 hour at 4°C, the supernatant was collected and incubated at 37°C to induce biphasic separation. The upper aqueous layer was re‐extracted as described above. The lipoglycans in the detergent layers were precipitated by the addition of nine volumes of ethanol (95%, 20°C). The precipitates were treated with proteinase K (#25530015, Invitrogen, Carlsbad, United States) for 2 hours at 60°C. The resultant solution containing ManLAM was dialysed and lyophilized. To purify ManLAM, HPLC was performed on an Agilent liquid chromatography system (Santa Clara, CA, USA) fitted with a Sephadex column (GE Healthcare) equilibrated with 0.2 mol/L NaCl, 0.25% deoxycholate, 1 mmol/L EDTA, 0.02% sodium azide and 10 mmol/L Tris (pH 8.0) at a flow rate of 1 mL/min.

### 2.3 B cell isolation

B cells were purified and isolated from murine splenocytes using CD19 positive magnetic‐activated cell sorting (#130‐052‐201, Miltenyi Biotec, Bergisch Gladbach, Germany). Briefly, the splenocytes were incubated with CD19 microbeads for 15 minutes at 4°C. CD19^+^ cells labelled with microbeads were separated from unlabelled cells via a column in the presence of a magnetic field. Purity of B cells was >95% as determined by FCM (Flow cytometry) using APC anti‐CD19 antibody (6D5, #115512).

### 2.4 FCM

The B cells isolated from the spleen or MLNs were stained with APC anti‐CD19 antibody (6D5, #115512) and then fixed and permeabilized with the fixation/ permeabilization buffer (Biolegend) according to the manufacturer's protocol. Permeabilized cells were stained with FITC anti‐IL‐10 antibody (JES5‐16E3, #505006). To identify CD4^+^ T cell polarization, APC anti‐CD3 antibody (17A2, #100236), FITC anti‐CD4 antibody (GK1.5, #100406), PE‐anti‐IL‐4 antibody (11B11, #504104), PE‐anti‐IFN‐γ antibody (XMG1.2, #505808) and PerCP‐Cy5.5 anti‐IL‐17A antibody (TC11‐18H10.1, #506920) were used for detection of intracellular cytokine expression. All antibodies used in FCM analysis were purchased from Biolegend and eBiosience (Thermo Fisher Scientific).

### 2.5 DSS‐induced murine IBD model

Two experiments were performed. To assess IL‐10 production by ManLAM‐treated B cells in vivo, B cells were isolated from splenocytes of WT/IL‐10^−/−^ mice and stimulated with ManLAM (10 ng/mL) for 12 hours.^16^ After washing, the ManLAM‐treated B cells were labelled with carboxyfluorescein succinimidyl ester (CFSE, 5 μmol/L, BD bioscience, #565082). The CESE‐labelled cells were suspended into PBS solution and adoptively transferred by *iv* (intravenous) injection into IL‐10^−/−^ mice (5** × **10^6^/100 μL PBS/mouse). Three days later, the B10 cell frequencies in spleen, MLNs and PBMCs (peripheral blood mononuclear cells) from the recipient mice were measured by FCM.

To assess the effects of the ManLAM‐induced B10 cells on murine IBD, ManLAM‐treated B cells (labelled with CFSE) were adoptively transferred into IL‐10^−/−^ mice (6 mice per group) on Day 3. The IL‐10^−/−^ mice were fed with 3% (w/v) DSS (#SKU 0216011080, MP Biomedicals, LCC, Solon, OH) in drinking water from Day 0 to Day 7, and then were followed by tap water as previously described.^19^ The body weight of mice was measured every day. On Day 9, the transferred B cells and B10 cell frequencies in MLNs were measured by FCM. Intestinal samples were harvested, and the lengths of colons were measured. Tissue samples from distal colon were prepared for histopathological analysis. IFN‐γ, IL‐4 and IL‐17A production by CD4^+^ T cells in spleen and MLNs were analysed by FCM.

### 2.6 Histopathological analysis

Intestinal samples were harvested from distal colon, fixed in 4% paraformaldehyde and embedded in paraffin for H&E (haematoxylin and eosin) staining. Histological IBD scoring was performed by a pathologist who was blinded to the treatment as follows: IBD scores were calculated as mucosal damage plus extension of the lesion. For the damage, 0 = none, 1 = loss of the basal 1/3 of the crypt, 2 = loss of the basal 2/3 of the crypt, 3 = loss of entire crypt but intact surface epithelial cells and 4 = loss of both the entire crypt and the surface epithelial cells. For the extension, 0 = none, 1 = focal, 2 = lesions involving 1/3 of the intestine, 3 = lesions involving 2/3 of the intestine and 4 = lesions involving the entire intestine.

### 2.7 Statistical analysis

Data are presented as mean ± SD and analysed by GraphPad Prism V 5.00 for Windows (GraphPad Software). Statistical significance was determined by ANOVA followed by Neuman‐Keuls post hoc test. *P <* .05 considered as statistically significant.

## 3 RESULTS

### 3.1 Transferred ManLAM‐treated B cells produce IL‐10 in the recipient mice

**Figure 1:**
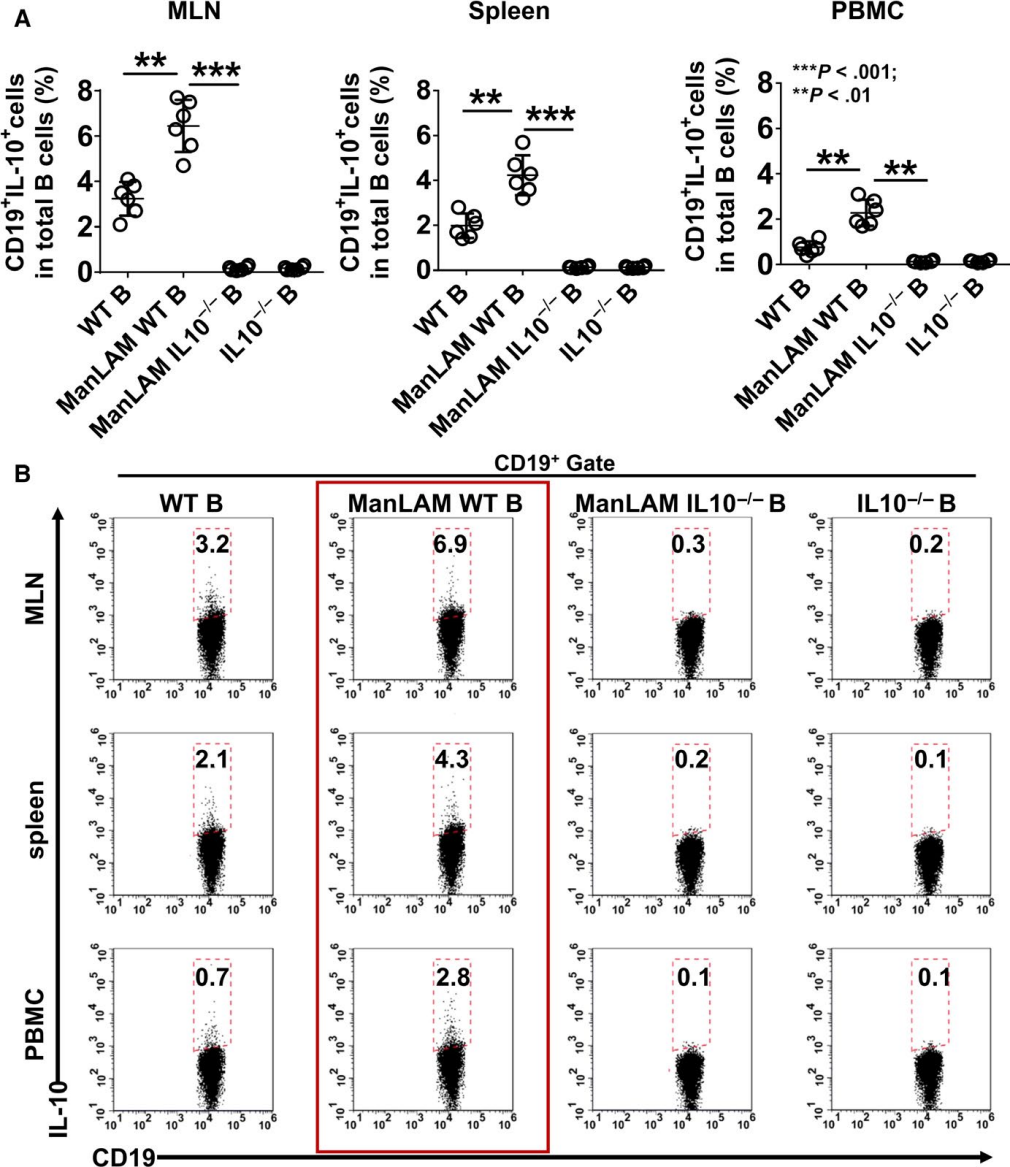
Transferred ManLAM‐treated B cells produce IL‐10 in the recipient mice. ManLAM‐treated B cells were adoptively transferred into IL‐10^−/−^ mice. Three days later, the B10 cell frequencies (among total B cells) in MLNs, spleen and PBMCs from the recipient mice were measured. A, Pooled data. B, Representative dot plots. The results are expressed as the means ± SD (n = 6). Statistical significance was determined by ANOVA followed by Neuman‐Keuls post hoc test

To investigate the role of ManLAM‐induced B10 cells in IBD model, we first assess whether ManLAM‐treated B cells produce IL‐10 in vivo. B cells were purified and isolated from splenocytes of WT/IL‐10^−/−^ mice. The B cells were stimulated with ManLAM for 12 hours.^16^ After washing the B cells, the cells were labelled with CFSE and adoptively transferred into IL‐10^−/−^ mice. Three days later, the B10 cell frequencies in MLNs, spleen and PBMCs (peripheral blood mononuclear cells) from the recipient mice were determined by FCM (Figure 1A,B). The higher frequencies of B10 cells in total B cells (2%‐7%) were observed in ManLAM‐WT B group compared with other groups (Figure 1A,B). The B cells from MLNs in ManLAM‐WT B group had the highest frequencies of B10 cells than those from spleen and PBMC, indicating that transferred ManLAM‐induced B10 cells could migrate to MLNs (Figure 1A,B).

### 3.2 ManLAM‐induced B10 cells alleviate the pathological symptoms in the DSS‐induced IBD in mice

Because transferred ManLAM‐WT B cells resulted in the highest frequencies of B10 cells in vivo, we hypothesized that ManLAM‐induced B10 cells would have a protective potential in IBD model. Toxic effects of DSS on the colonic epithelium stimulate an inflammatory response in intestinal tract.^20^, ^21^ Therefore, we used DSS to induce acute colitis in mice.

**Figure 2:**
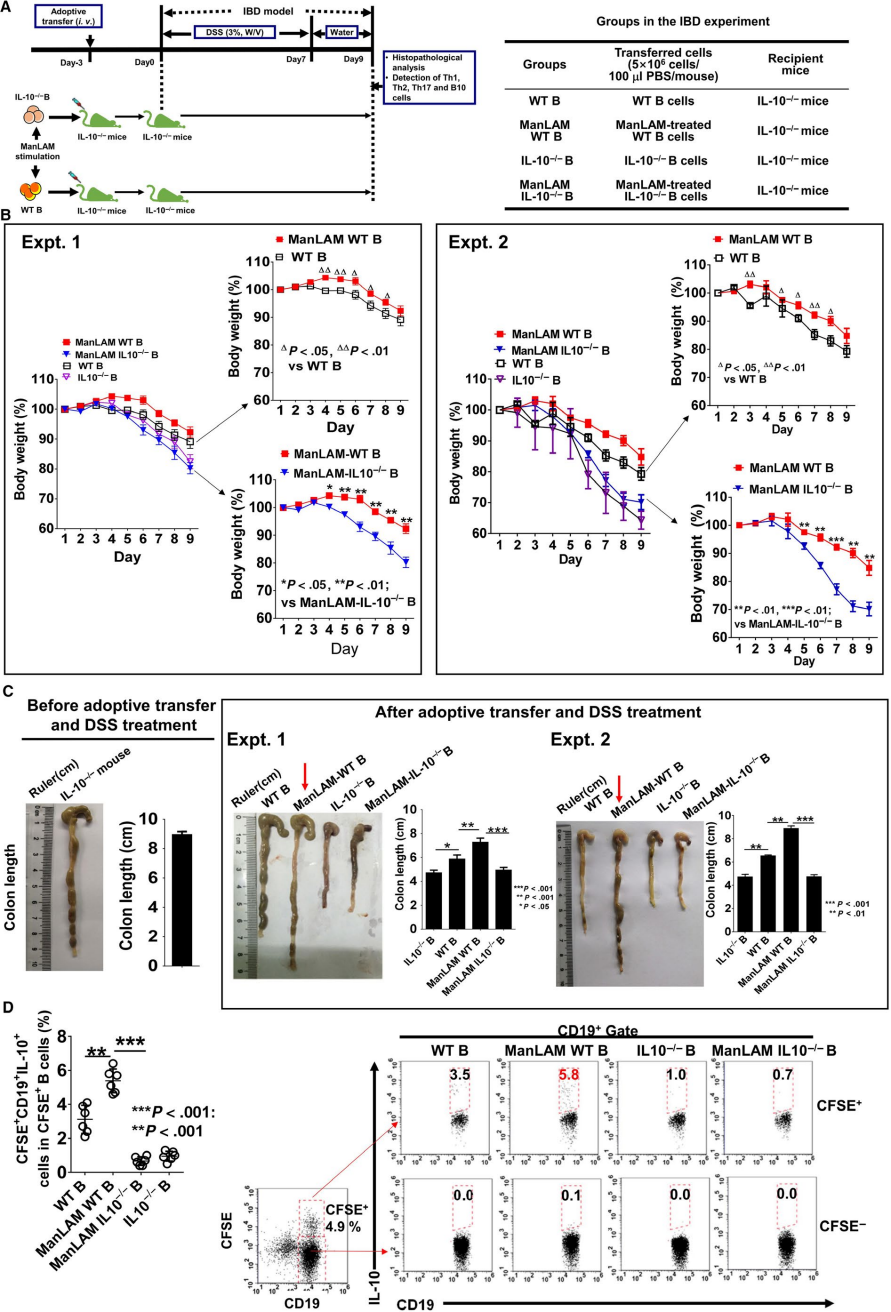
The mice transferred with ManLAM‐treated WT B cells were more resistant to dextran sodium sulphate (DSS) treatment. ManLAM‐treated WT/IL‐10^−/−^ B cells were transferred into the IL‐10^−/−^ mice. The recipient mice were treated with DSS for acute inflammatory bowel disease (IBD) development. A, Schematic diagram. B, Mouse body weight was monitored daily, and body weight (%) was expressed as percentage of body weight at Day 1. C, Macroscopic changes and colon length on Day 3 (left panel) and Day 9 (Right panel). D, The B10 cell frequencies in CFSE^+^ transferred B cells in MLNs (from Expt. 1) were measured by flow cytometry on Day 9. The data are presented as mean ± SD (n = 6). Statistical significance was determined by ANOVA followed by Neuman‐Keuls post hoc test

On Day 3, ManLAM‐treated B cells were adoptively transferred into IL‐10^−/−^ mice. The recipient mice were then fed with 3% (w/v) DSS in drinking water for 7 days followed by tap water (Figure 2A). After treatment with DSS, we monitored changes in body weight of the mice (Figure 2B). Three‐four days after DSS treatment, the body weight of the mice in all groups began to decrease (Figure 2B). The loss of body weight in ManLAM‐WT B group was significantly slower than that in ManLAM‐IL‐10^−/−^ B group (Figure 2B), suggesting that IL‐10 produced by ManLAM‐treated WT B cells might attenuate the DSS‐induced acute colitis in mice. The significant differences in the loss of body weight between ManLAM‐treated WT B group and WT B group were also observed from Day 4 to Day 8, indicating that the ManLAM treatment of B cells has therapeutic effects on murine IBD (Figure 2B).

Colitis increases oedema and shortens the overall colon length. Colon shortening is used as a marker of colon inflammation.^22^ As shown in Figure 2C left panel, the average length of normal IL‐10^−/−^ mouse colon before adoptive transfer and DSS treatment was 8.95 cm (95% CI: 8.42‐9.48). After DSS treatment (on Day 9), the colon lengths in all experimental groups were reduced probably because of DSS‐induced intestinal injury (Figure 2C right panel). Consistent with the body weight loss, colon shortening was reduced in ManLAM‐WT B group compared with ManLAM‐IL‐10^−/−^ B group, demonstrating that ManLAM‐induced B10 cells alleviate inflammation in colon (Figure 2C right panel). There was significant difference (**P < .01) in colon length between ManLAM‐WT B group and WT B group, while transferring ManLAM‐treated IL‐10^−/−^ B cells did not cause the reduction of colon length compared with IL‐10^−/−^ B cells (Figure 2C right panel).

On Day 9, the transferred B cells and B10 cell frequencies in MLNs were measured by FCM. As shown in Figure 2D, transferred CFSE^+^ B cells made up 4.9% of all B cells in the mice. The highest B10 cell frequency of CFSE^+^ B cells was found in ManLAM‐WT B group, while the CFSE^‐^ B cells of the recipient mice did not produce IL‐10 (Figure 2D). These results demonstrated that the transferred ManLAM‐treated B cells produce IL‐10 in the intestinal tract. Taken together, our results demonstrate that adoptive transfer of ManLAM‐induced B10 cells alleviates the pathological symptoms in the DSS‐induced IBD in mice.

### 3.3 ManLAM‐induced B10 cells ameliorates intestinal inflammation in the mice

**Figure 3:**
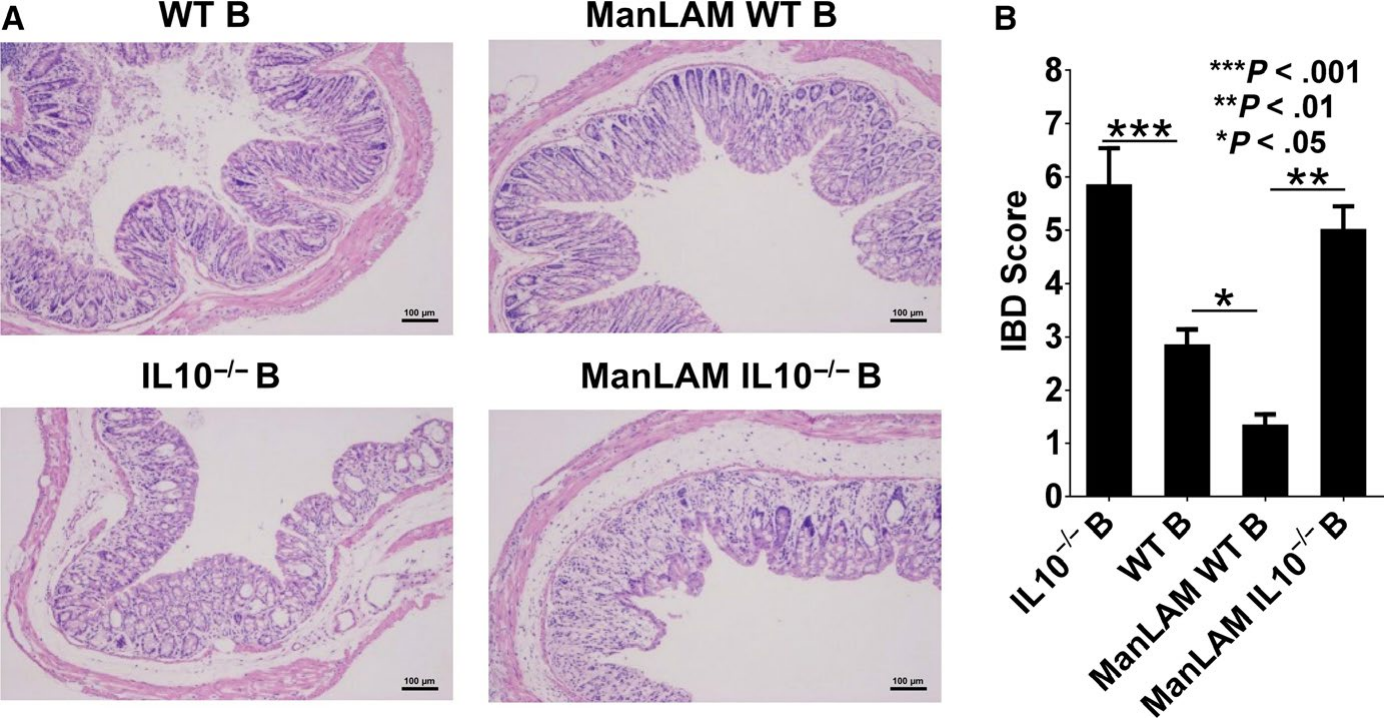
ManLAM‐treated WT B cell alleviated the pathological symptoms in the dextran sodium sulphate (DSS)‐induced inflammatory bowel disease (IBD) in mice. A, Histological analysis after the DSS administration in mice on Day 9. Representative histological images of distal colon sections stained with H&E. Scale bars, 100 μm. B, Histologic inflammatory score. The results are expressed as the mean ± SD (n = 6). Statistical significance was determined by ANOVA followed by Neuman‐Keuls post hoc test

To further assess the effects of ManLAM‐induced B10 cells on intestinal inflammation, histological analysis of the mouse colon tissue was performed using H&E staining. As shown in Figure 3A, colitis in IL‐10^−/−^ B and ManLAM‐ IL‐10^−/−^ B group was characterized by a loss of crypts, infiltration of inflammation cells into the mucosa and submucosa, oedema of submucosa, erosion and ulceration. These histological changes in the murine DSS‐induced IBD were showed some features of human Crohn's disease which was characterized by transmural inflammation with disseminated lymphoid follicles and focal lesions. However, attenuated colitis severity was observed in ManLAM‐WT B group with markedly reduced colonic crypt damage, fewer inflammatory infiltrates (Figure 3A). Transferring WT B cells also slightly alleviated inflammation and damage in the intestines (Figure 3A).

The damage to intestinal mucosa was then graded through histological scoring. As shown in Figure 3B, compared with the mice treated with ManLAM‐WT B cells, colitis scores were significantly higher in the mice subjected to the treatment with WT B, IL‐10^−/−^ B and ManLAM‐treated IL‐10^−/−^ B cells, mostly due to typical inflammatory changes including goblet cell loss and crypt damage. There was significantly higher score in WT B group compared with ManLAM‐WT B group (*P < .05), suggesting that the higher level of IL‐10 produced by ManLAM‐treated B cells facilitated to ameliorate intestinal inflammation in the mice with IBD (Figure 3B).

### 3.4 ManLAM‐induced B10 cells modulate intestinal inflammation response via hindering Th1 polarization but promoting Th2 polarization

**Figure 4:**
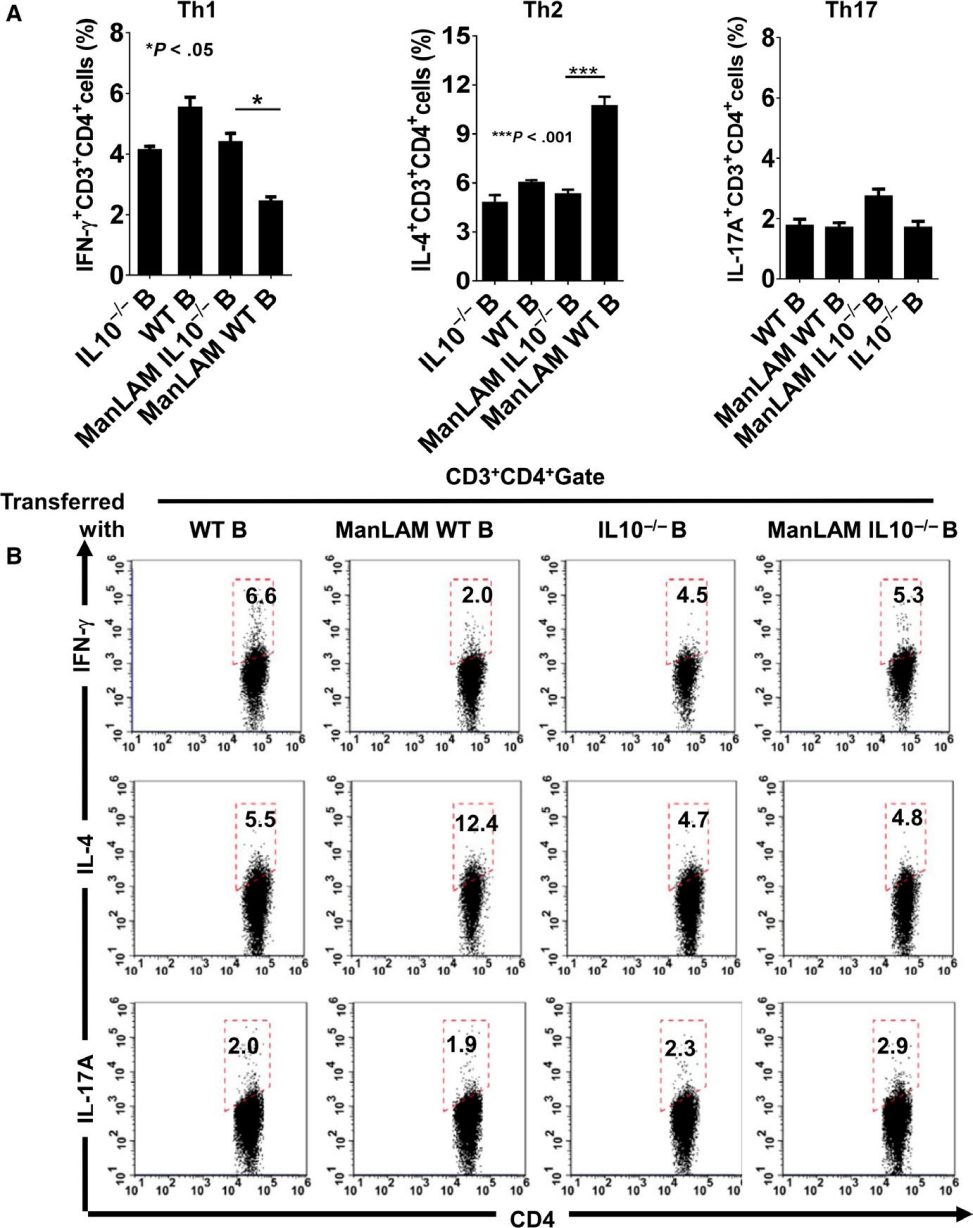
The IFN‐γ, IL‐4 and IL‐17A productions by splenic CD4^+^T cells in the murine inflammatory bowel disease (IBD) model were determined by flow cytometry (FCM). On Day 9, the recipient mice were sacrificed and IFN‐γ, IL‐4 and IL‐17A productions of splenic CD4^+^ T cells were analysed by FCM. A, Pooled data. B, Representative dot plots. The results are expressed as the means ± SD (n = 6). Statistical significance was determined by ANOVA followed by Neuman‐Keuls post hoc test

In Crohn's disease, the major cytokines arise from Th1 and Th17 CD4^+^ T cell differentiation.^23^ To elucidate the mechanism for ManLAM‐induced B10 cells alleviating the pathological symptoms and intestinal inflammation in DSS‐induced colitis in mice, we investigate the effects of these B10 cells on CD4^+^ T cell polarization. As shown in Figure 4A,B, the cytokine productions of IFN‐γ, IL‐4 and IL‐17A by splenic CD4^+^ T cells were determined by FCM. Compared with ManLAM‐treated IL‐10^−/−^ B cells, transferring ManLAM‐treated WT B cells significantly suppressed IFN‐γ production by CD4^+^ T cells but enhanced IL‐4 production (Figure 4A,B). These results demonstrated that ManLAM‐induced B10 cells hinders the Th1 polarization but promotes Th2 polarization. There were no significant differences in IL‐17A production by splenic CD4^+^ T cells among the groups, indicating that ManLAM‐induced B10 had no effects on Th17 differentiation (Figure 4A,B).

**Figure 5:**
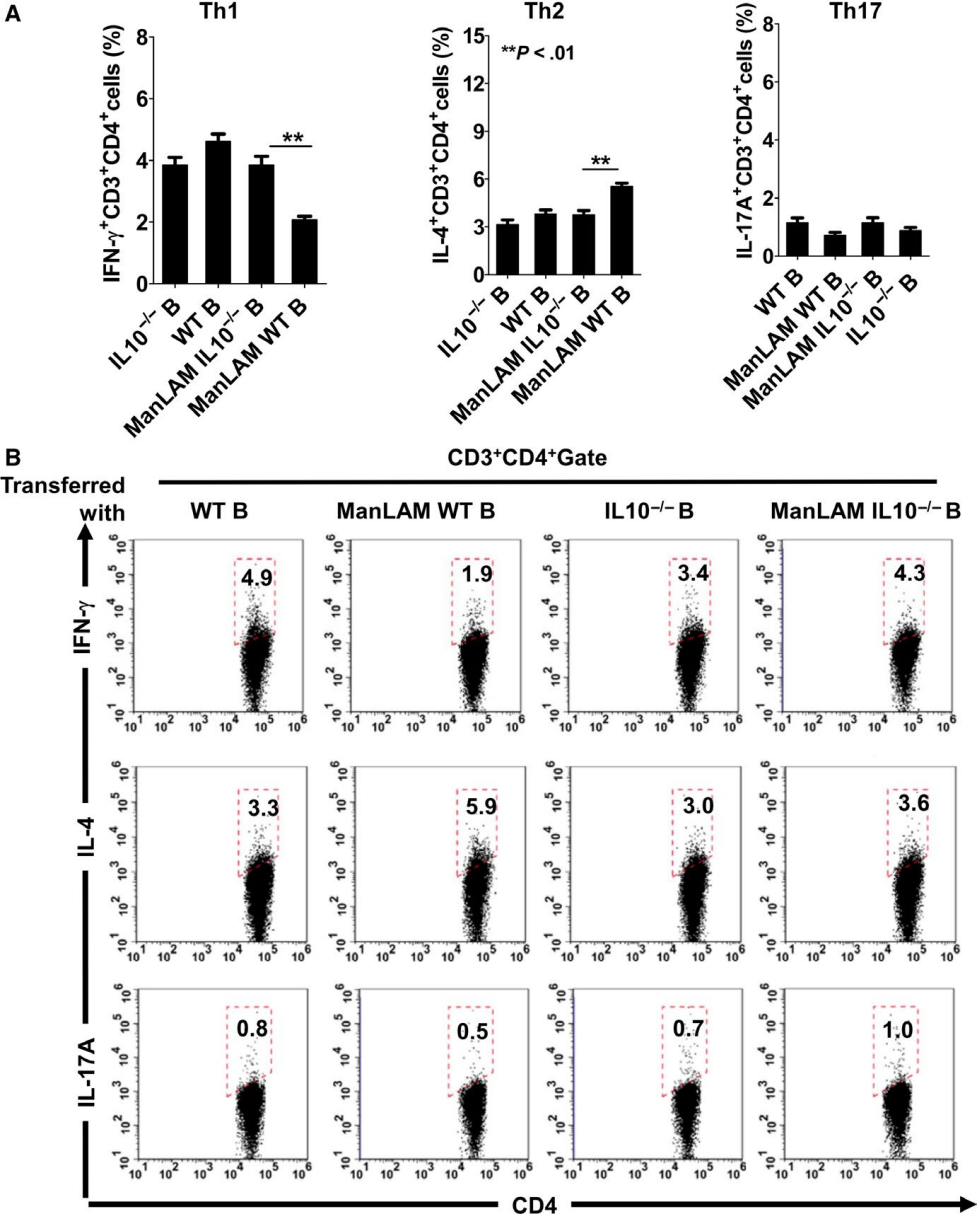
The IFN‐γ, IL‐4 and IL‐17A productions by CD4^+^T cells in MLN in the murine IBD model were determined by flow cytometry (FCM). On Day 9, the recipient mice were sacrificed and IFN‐γ, IL‐4 and IL‐17A productions of CD4^+^ T cells in MLN were analysed by FCM. A, Pooled data. B, Representative dot plots. The results are expressed as the means ± SD (n = 6). Statistical significance was determined by ANOVA followed by Neuman‐Keuls post hoc test

The Th1/ Th2 polarization in MLN was also determined by FCM (Figure 5A,B). Consistent with the results from splenocytes, the lowest level of IFN‐γ production by CD4^+^ T cells and the highest level of IL‐4 production were observed in ManLAM‐WT B groups. These results demonstrated that the ManLAM‐induced B10 cells modulate the Th1/Th2 response in the intestine.

Moreover, transferring ManLAM‐treated WT B cells decreased IFN‐γ production but increased IL‐4 production by CD4^+^ T cells compared with WT B group, probably because ManLAM induced higher frequencies of B10 cells (Figure 4 and Figure 5). Collectively, these results suggest ManLAM‐induced B10 cells downregulate Th1 polarization and decrease susceptibility to DSS‐induced IBD in mice.

## 4 DISCUSSION

IL‐10 is an important anti‐inflammatory cytokine, which suppresses pro‐inflammatory cytokine production and regulates Th1/Th2 polarization.^24^, ^25^ Besides dissecting the role of Tregs in protection against IBD,^26^ several studies have shown that IL‐10 production by B cells is associated with reducing severity of IBD disease.^8^, ^9^, ^10^, ^11^, ^12^ In patients with Crohn's disease, CpG DNA‐induced IL‐10 production by B cells was significantly decreased.^11^ In murine IBD model, B10 cells modulate IFN‐γCD4^+^ T cell number, neutrophil infiltration and pro‐inflammatory cytokine production during colitis onset.^8^ These B10 cells were characterized as CD1d^high^ B1 cells.^10^, ^12^

Recently, we have reported that B10 cells were induced by ManLAM, a mycobacterial lipoglycan.^16^ In the current study, we aim to determine whether ManLAM could protect mice from IBD via IL‐10 production by B cells. WT mice have a low level of B10 cells. To determine the accurate function of transferred ManLAM‐induced B10 cells, the IL‐10^−/−^ mice were employed as recipients in IBD model, in which only transferred ManLAM‐treated B cells produced the IL‐10 and the recipient mice themselves had no B10 cell background. We demonstrate B10 cells induced by ManLAM regulate Th1/Th2 cytokine balance and decrease severity of IBD compared with ManLAM‐IL‐10^−/−^ B cell group, suggesting that ManLAM‐treated B cells have a preventive effect on colitis and this beneficial effect is mediated via IL‐10 production. To the best of our knowledge, this is the first report about the potential protective roles of ManLAM‐treated B cells against mucosal inflammatory disorders in the gut. However, IBD induced in WT mouse recipients might more accurately reflect the disruption of intestinal immune homeostasis. The protective effects of ManLAM‐induced B10 cells on IBD should be evaluated in WT mouse recipients in future study.

In the current study, we did not extract B cells from MLN tissue for adoptive transfer. Instead, we extracted B cells from spleens because we could get abundant splenic B cells for adoptive transfer experiment. In our experiment, adoptively transferred B cells were detected in MLN tissue of the recipient mice on Day 9 (Figure 2D), demonstrating that transferred B10 cells can migrate into MLN tissue to regulate Th1/Th2 cytokine balance and decrease severity of IBD. Other study also reported that transferring B10 cells from mouse peripheral blood effectively blocks the development of colitis in IL‐10^−/−^ mice.^27^ The results from our current study and other reports indicate that transferring B10 cells isolated from non‐MLN tissues can alleviate the mucosal inflammatory disorders in the gut.

B10 cells have been demonstrated to be potent regulators of allergic and autoimmune disease, transplant rejection, infection and cancer.^28^ It has been reported that the adoptive transfer of B10 cells induced by LPS (lipopolysaccharide, TLR4 agonist) or CpG ODN1826 (oligodeoxynucleotide, TLR9 agonist) reduced the severity of EAE (experimental autoimmune encephalomyelitis) via suppressing IFN‐γ and IL‐17 production by CD4^+^ T cells.^29^, ^30^ In the current study, ManLAM‐induced B10 cells suppressed the IFN‐ γ production by CD4^+^ T cells, but did not change the IL‐17 production (Figures 4 and 5). These results were consistent with our previous report that ManLAM‐induced B10 cells did not alter the Th17 polarization during mycobacterial infection.^16^ B10 cells represent a population of B cells with diverse phenotype and function.^28^ ManLAM induces IL‐10 production by B cells via TLR2‐signalling pathway,^16^ so the ManLAM‐induced B10 cell population might be different with the B10 cells induced by TLR4 agonist and TLR9 agonist, resulting in the functional differences of the B10 cells. Importantly, adoptive transfer of ManLAM‐induced B10 cells alleviated the pathological symptoms and intestinal inflammation in the mice with colitis (Figures 2 and 3), demonstrating that B10 cells alone could contribute to reversing the unbalance of CD4^+^ T cells.

It has been reported that B10 cells are a significant source of IL‐10 in WT mice, and IL‐10 production from regulatory B10 cells ameliorates symptoms and regulates DSS‐induced intestinal injury in IL‐10‐sufficient mice.^10^ Although IL10^−/−^ mice have more severe colitis than WT mice, IL‐10^−/−^ mice were wildly used in IBD model to investigate the roles of IL‐10 and IL‐10‐producing cells.^27^, ^31^ In the current study, we assessed the protective effects of ManLAM‐induced B10 cells. Consistent with our results, Mishima et al also report that microbiota‐induced intestinal B10 cells ameliorated chronic T cell‐mediated colitis in IL‐10 deficient background mice.^31^ However, since all experiments in the current study are carried out in IL10^−/−^ mice, it is not clear whether ManLAM‐treated B cells would also play a role in IL10‐sufficient animals. A more accurate assessment of roles of ManLAM‐treated B cells should be performed in IBD model of IL10‐sufficient mice.

We assessed the IFN‐γ, IL‐4 and IL‐17A production by CD4^+^T cells in spleens and MLN, and we found that ManLAM‐induced B10 cells suppress the Th1 polarization. However, the T cells exert their disease‐relevant effector function in the tissue rather than in secondary lymphoid organs. Assessment of the effects of ManLAM‐induced B10 cells on T cell polarization among tissue‐resident effector T cells in the intestinal lamina propria should be performed in future study for more accurately revealing the mechanism by which the B10 cells decrease colitis severity in the mice.

Additionally, ManLAM, derived from *Mycobacterium*, can be recognized by several pathogen pattern receptors, including TLR2 and mannose receptor (MR). In the current study, we demonstrate that ManLAM‐treated B cells suppress Th1 polarization via IL‐10. IL‐10 production by ManLAM‐treated B cells is predominantly mediated TLR2‐signalling pathway.^16^ We hypothesize that ManLAM might be recognized by other receptors on B cells and employ other mechanism to ameliorate IBD. For example, it has been reported that mannose‐binding lectin (MBL) or mannose receptor (MR), which are highly expressed in the intestinal epithelial cells, is required for intestinal homeostasis.^32^ The impairment in MBL function is associated with IBD.^33^ Whether ManLAM triggers the MR (or MBL)‐related signalling pathway in B cells to promote the protection cells against IBD should be investigated in future study.

The exact pathogenesis of IBD remains unknown, but multiple inflammatory pathways and cellular and microbiota contributions have been reported. Although novel treatments and strategies based on these observations have been developed, there are still some limitations in IBD therapy.^34^ In this study, our results demonstrate that ManLAM‐induced B10 cells decrease susceptibility to DSS‐induced IBD in mice and indicate that agents with the ability to induce B10 cells might be candidates for IBD treatment, like ManLAM.

## CONFLICT OF INTEREST

The authors declared no conflict of interest.

## AUTHORS’ CONTRIBUTIONS

Q. Pan conceived and designed the experiments. C. Yuan, X. Li, L. Luo, Y. Wang, D. Zhang performed the experiments. K‐L. Zhou analysed the data. C. Yuan and X. Li wrote the manuscript. X‐L. Zhang and Q. Pan revised the manuscript.

## References

[1] Yanaba K , Bouaziz JD , Haas KM , Poe JC , Fujimoto M , Tedder TF . A regulatory B cell subset with a unique CD1d(hi )CD5(+) phenotype controls T cell‐dependent inflammatory responses. Immunity. 2008;28(5):639‐650. 18482568 10.1016/j.immuni.2008.03.017

[2] Yoshizaki A , Miyagaki T , DiLillo DJ , et al. Regulatory B cells control T‐cell autoimmunity through IL‐21‐dependent cognate interactions. Nature. 2012;491(7423):264‐268. 23064231 10.1038/nature11501 PMC3493692

[3] Kalampokis I , Yoshizaki A , Tedder TF . IL‐10‐producing regulatory B cells (B10 cells) in autoimmune disease. Arthritis Res Ther. 2013;15(Suppl 1):S1. 10.1186/ar3907 PMC3624502 23566714

[4] Tedder TF . B10 cells: a functionally defined regulatory B cell subset. J Immunol. 2015;194:1395‐1401. 25663677 10.4049/jimmunol.1401329

[5] Nishida A , Inoue R , Inatomi O , Bamba S , Naito Y , Andoh A . Gut microbiota in the pathogenesis of inflammatory bowel disease. Clin J Gastroenterol. 2018;11:1‐10. 29285689 10.1007/s12328-017-0813-5

[6] Cammarota G , Ianiro G , Cianci R , Bibbo S , Gasbarrini A , Curro D . The involvement of gut microbiota in inflammatory bowel disease pathogenesis: potential for therapy. Pharmacol Ther. 2015;149:191‐212. 25561343 10.1016/j.pharmthera.2014.12.006

[7] Nell S , Suerbaum S , Josenhans C . The impact of the microbiota on the pathogenesis of IBD: lessons from mouse infection models. Nat Rev Microbiol. 2010;8:564‐577. 20622892 10.1038/nrmicro2403

[8] Maseda D , Candando KM , Smith SH , et al. Peritoneal cavity regulatory B cells (B10 cells) modulate IFN‐γ(+)CD4(+)T cell numbers during colitis development in mice. J Immunol. 2013;191:2780‐2795. 23918988 10.4049/jimmunol.1300649 PMC3770313

[9] Wei B , Velazquez P , Turovskaya O , et al. Mesenteric B cells centrally inhibit CD4(+)T cell colitis through interaction with regulatory T cell subsets. Proc Natl Acad Sci USA. 2005;102:2010‐2015. 15684084 10.1073/pnas.0409449102 PMC548553

[10] Yanaba K , Yoshizaki A , Asano Y , Kadono T , Tedder TF , Sato S . IL‐10‐producing regulatory B10 cells inhibit intestinal injury in a mouse model. Am J Pathol. 2011;178:735‐743. 21281806 10.1016/j.ajpath.2010.10.022 PMC3069829

[11] Oka A , Ishihara S , Mishima Y , et al. Role of regulatory B cells in chronic intestinal inflammation: association with pathogenesis of Crohn's disease. Inflamm Bowel Dis. 2014;20:315‐328. 24390063 10.1097/01.MIB.0000437983.14544.d5

[12] Mizoguchi A , Mizoguchi E , Takedatsu H , Blumberg RS , Bhan AK . Chronic intestinal inflammatory condition generates IL‐10‐producing regulatory B cell subset characterized by CD1d upregulation. Immunity. 2002;16:219‐230. 11869683 10.1016/s1074-7613(02)00274-1

[13] Kowalewicz‐Kulbat M , Locht C . BCG and protection against inflammatory and auto‐immune diseases. Expert Rev Vaccines. 2017;16:1‐10. 10.1080/14760584.2017.1333906 28532186

[14] Lagranderie M , Kluge C , Kiefer‐Biasizzo H , et al. *Mycobacterium bovis* Bacillus Calmette‐Guerin killed by extended freeze‐drying reduces colitis in mice. Gastroenterology. 2011;141:642‐652.e4. 21683076 10.1053/j.gastro.2011.05.002

[15] Villumsen M , Jess T , Sorup S , et al. Risk of inflammatory bowel disease following Bacille Calmette‐Guerin and smallpox vaccination: a population‐based Danish case‐cohort study. Inflamm Bowel Dis. 2013;19:1717‐1724. 23624886 10.1097/MIB.0b013e318281f34e

[16] Yuan C , Qu Z‐L , Tang X‐L , et al. Mycobacterium tuberculosis Mannose‐Capped Lipoarabinomannan Induces IL‐10‐Producing B Cells and Hinders CD4+Th1 Immunity. iScience. 2019;11:13‐30. 30572206 10.1016/j.isci.2018.11.039 PMC6299163

[17] Kühn R , Löhler J , Rennick D , Rajewsky K , Müller W . Interleukin‐10‐deficient mice develop chronic enterocolitis. Cell. 1993;75(2):263‐274. 8402911 10.1016/0092-8674(93)80068-p

[18] Sun X , Pan Q , Yuan C , et al. A single ssDNA aptamer binding to mannose‐capped lipoarabinomannan of Bacillus Calmette‐Guerin enhances immunoprotective effect against tuberculosis. J Am Chem Soc. 2016;138:11680‐11689. 27529508 10.1021/jacs.6b05357

[19] Yang Y‐F , Zhou Y‐D , Hu J‐C , et al. Ficolin‐A/2, acting as a new regulator of macrophage polarization, mediates the inflammatory response in experimental mouse colitis. Immunology. 2017;151:433‐450. 28380665 10.1111/imm.12741 PMC5506452

[20] Eichele DD , Kharbanda KK . Dextran sodium sulfate colitis murine model: An indispensable tool for advancing our understanding of inflammatory bowel diseases pathogenesis. World J Gastroenterol. 2017;23:6016‐6029. 28970718 10.3748/wjg.v23.i33.6016 PMC5597494

[21] Zhao H , Zhang H , Wu H , et al. Protective role of 1,25(OH)_2_ vitamin D_3_ in the mucosal injury and epithelial barrier disruption in DSS‐induced acute colitis in mice. BMC Gastroenterol. 2012;12:57. 22647055 10.1186/1471-230X-12-57 PMC3464614

[22] Whittem CG , Williams AD , Williams CS . Murine colitis modeling using dextran sulfate sodium (DSS). J Vis Exp. 2010 pii:1652. 10.3791/1652 PMC2841571 20087313

[23] Strober W , Fuss IJ . Proinflammatory cytokines in the pathogenesis of inflammatory bowel diseases. Gastroenterology. 2011;140:1756‐1767. 21530742 10.1053/j.gastro.2011.02.016 PMC3773507

[24] Asadullah K , Sterry W , Volk HD . Interleukin‐10 therapy–review of a new approach. Pharmacol Rev. 2003;55:241‐269. 12773629 10.1124/pr.55.2.4

[25] Woodfolk JA . Selective roles and dysregulation of interleukin‐10 in allergic disease. Curr Allergy Asthma Rep. 2006;6:40‐46. 16476193 10.1007/s11882-006-0008-5

[26] Workman CJ , Szymczak‐Workman AL , Collison LW , Pillai MR , Vignali DA . The development and function of regulatory T cells. Cell Mol Life Sci. 2009;66:2603‐2622. 19390784 10.1007/s00018-009-0026-2 PMC2715449

[27] Sattler S , Ling G‐S , Xu D , et al. IL‐10‐producing regulatory B cells induced by IL‐33 (Breg(IL‐33)) effectively attenuate mucosal inflammatory responses in the gut. J Autoimmun. 2014;50:107‐122. 24491821 10.1016/j.jaut.2014.01.032 PMC4012142

[28] Candando KM , Lykken JM , Tedder TF . B10 cell regulation of health and disease. Immunol Rev. 2014;259:259‐272. 24712471 10.1111/imr.12176 PMC4049540

[29] Hong J , Fang J , Lan R , et al. TLR9 mediated regulatory B10 cell amplification following sub‐total body irradiation: implications in attenuating EAE. Mol Immunol. 2017;83:52‐61. 28110075 10.1016/j.molimm.2017.01.011

[30] Matsushita T , Horikawa M , Iwata Y , Tedder TF . Regulatory B cells (B10 cells) and regulatory T cells have independent roles in controlling experimental autoimmune encephalomyelitis initiation and late‐phase immunopathogenesis. J Immunol. 2010;185:2240‐2252. 20624940 10.4049/jimmunol.1001307 PMC3717968

[31] Mishima Y , Oka A , Liu B , et al. Microbiota maintain colonic homeostasis by activating TLR2/MyD88/PI3K signaling in IL‐10‐producing regulatory B cells. J Clin Invest. 2019;129(9):3702‐3716. 31211700 10.1172/JCI93820 PMC6715367

[32] Choteau L , Parny M , François N , et al. Role of mannose‐binding lectin in intestinal homeostasis and fungal elimination. Mucosal Immunol. 2016;9:767‐776. 26442658 10.1038/mi.2015.100

[33] Choteau L , Vasseur F , Lepretre F , et al. Polymorphisms in the mannose‐binding lectin gene are associated with defective mannose‐binding lectin functional activity in Crohn's disease patients. Sci Rep. 2016;6:29636. 27404661 10.1038/srep29636 PMC4940739

[34] Weisshof R , El Jurdi K , Zmeter N , Rubin DT . Emerging therapies for inflammatory bowel disease. Adv Ther. 2018;35:1746‐1762. 30374806 10.1007/s12325-018-0795-9 PMC6224002

